# Effect of cardiopulmonary bypass on coagulation factors II, VII and X in a primate model: an exploratory pilot study

**DOI:** 10.1093/icvts/ivad194

**Published:** 2023-11-28

**Authors:** Tomonari Shimoda, Chang Liu, Bryan J Mathis, Yukinobu Goto, Naohide Ageyama, Hideyuki Kato, Muneaki Matsubara, Tomohiro Ohigashi, Masahiko Gosho, Yasuyuki Suzuki, Yuji Hiramatsu

**Affiliations:** Department of Cardiovascular Surgery, Institute of Medicine, University of Tsukuba, Ibaraki, Japan; Department of Cardiovascular Surgery, Institute of Medicine, University of Tsukuba, Ibaraki, Japan; International Medical Center, University of Tsukuba Hospital, Ibaraki, Japan; Department of Thoracic Surgery, Institute of Medicine, University of Tsukuba, Ibaraki, Japan; Tsukuba Primate Research Center, National Institute of Biomedical Innovation, Tsukuba, Ibaraki, Japan; Department of Cardiovascular Surgery, Institute of Medicine, University of Tsukuba, Ibaraki, Japan; Department of Cardiovascular Surgery, Institute of Medicine, University of Tsukuba, Ibaraki, Japan; Tsukuba Clinical Research & Development Organization, University of Tsukuba Hospital, Ibaraki, Japan; Department of Biostatistics, Institute of Medicine, University of Tsukuba, Ibaraki, Japan; Department of Cardiovascular Surgery, Institute of Medicine, University of Tsukuba, Ibaraki, Japan; Department of Cardiovascular Surgery, Institute of Medicine, University of Tsukuba, Ibaraki, Japan

**Keywords:** Cardiopulmonary bypass, Coagulation factors, Postoperative bleeding

## Abstract

**OBJECTIVES:**

The use of cardiopulmonary bypass (CPB) in cardiac surgery is a major risk factor for postoperative bleeding. We hypothesized that consumptive coagulopathy and haemodilution influence the coagulation factors; therefore, we aimed to estimate the activity profiles of coagulation factors II, VII and X during CPB circulation.

**METHODS:**

A 120-min bypass was surgically established in cynomolgus monkeys (*n* = 7). Activities of coagulation factors II, VII and X were measured at 6 time points during the experiment (baseline, 0, 30, 60, 120 min of bypass and 60 min after bypass). To assess the influence of consumptive coagulopathy, the values were adjusted for haemodilution using the haematocrit values. Data were expressed as mean (standard deviation).

**RESULTS:**

Activities of coagulation factors decreased during the experiment. In particular, the activities for II, VII and X were decreased the most by 44.2% (5.0), 61.4% (4.3) and 49.0% (3.7) at 30 min following CPB initiation (*P* < 0.001, *P* < 0.001 and *P* < 0.001, respectively). Following adjustments for haemodilution, change magnitudes lessened but remained significant for factor VII. The adjusted concentration of factor VII was observed to decrease from the baseline to the initiation of bypass circulation.

**CONCLUSIONS:**

In conclusion, coagulation factor II, VII and X concentrations decreased during CPB. Following adjustment for haemodilution, a decrease in concentration was observed with factor VII.

**Figure:**
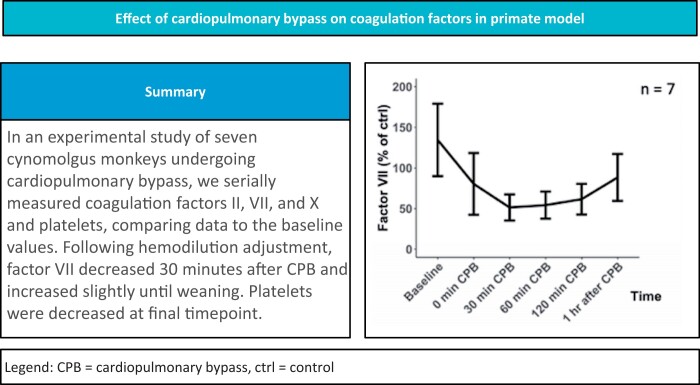

## INTRODUCTION

Cardiopulmonary bypass (CPB) is an underlying factor for coagulopathy following cardiac surgeries due to major blood loss that often necessitates allogenic blood transfusions and reoperations. Two major mechanisms are theorized to contribute to CPB-mediated coagulopathy, namely consumptive coagulopathy and haemodilution due to priming solution.

During CPB, mechanical pumps move blood over non-biological surfaces, including the oxygenator, in a non-laminar fashion [1]. This repetitive action triggers the intrinsic coagulation cascade, the disruption of which was long believed to play a major role in consumptive coagulopathy during CPB [1]. Recently, a shift in the paradigm indicates that extrinsic pathway disruption is also an important part of CPB-mediated coagulopathy [2]. The extrinsic pathway, activated by tissue factors released from the surgical wounds, causes thrombotic stimulus via activation of factor VII [2]. Such consumptive coagulopathy can be further potentiated by haemodilution due to the priming solution within the CPB circuit.

Coagulation factors II, VII and X are of particular importance as they play a key role in both intrinsic and extrinsic pathways [3]. Although several clinical studies have reported associations between coagulation factor activity and CPB, such data are influenced by a multitude of factors, including intraoperative transfusion, blood loss and diversity in operation types. To our knowledge, no current studies have evaluated changes in coagulation factor activities during CPB circulation in a standardized experimental setting.

Our group previously established a primate model of CPB circulation with emphasis on platelets and coagulation cascades [4]. We hypothesized that coagulation factor activities in this model would decrease during CPB due to consumptive coagulopathy. Therefore, we aimed to precisely profile coagulation factors II, VII and X during CPB circulation.

## MATERIALS AND METHODS

### Ethical statement

Male cynomolgus monkeys (*Macaca fascicularis*) born in the Tsukuba Primate Center were used as the nonhuman primate model in this study. The study strictly adhered to the Rules for Animal Care and Management of the Tsukuba Primate Center [5] and to the Guiding Principles for Animal Experiments Using Nonhuman Primates formulated by the Primate Society of Japan [6]. The study also strictly adhered to the Animal Research: Reporting of In Vivo Experiments guidelines 2.0 [7]. The study protocols, including ethical principles of laboratory animal care, were approved by the Animal Welfare and Animal Care Committee of the National Institute of Infectious Diseases (Tokyo, Japan) (Approval # DSR03-4, 8 April 2021) and by the Animal Experimentation Committee of the University of Tsukuba (Approval # 21-387, 1 June 2021).

### Experimental design

Changes in the activity of coagulation factors II, VII and X and concentration of platelets during CPB circulation were evaluated using our established nonhuman primate model as previously described [4]. We did not perform a power calculation in advance because it was an exploratory study. The sample size was small due to ethical and practical reasons*.* No concurrent control group was used as the data were compared to baseline. Briefly, median sternotomy and pericardial incision were performed. Systemic heparinization (250 U/kg) with activated clotting time ≥480 s was achieved, and standard CPB was established with ascending aortic cannulation for arterial inflow and superior and inferior vena cava cannulations for venous outflow in 7 monkeys for 2 h. The circuit of the CPB was assembled with polytetrafluoroethylene tubing (Mera Exceline; Senko Medical Instrument Mfg., Tokyo, Japan) and an infant-type membrane oxygenator (HPO-06RHF-C; Senko Medical Instrument Mfg.), as well as an arterial filter (HAF-C1; Senko Medical Instrument Mfg.) and a roller pump (HAD-11; Senko Medical Instrument Mfg.). An open circuit extracorporeal circulation model was used to better represent current clinical practice. The activated clotting time level was maintained at above 480 s. At 30 min after CPB initiation, tourniquets at the superior and inferior vena cava were tightened and complete extracorporeal circulation was established to prevent systematic blood from entering the right atrium. A right atrial incision was performed and coronary sinus blood was intentionally allowed to overflow into the pericardial cavity. Throughout this phase, the continuous application of a pump sucker facilitated blood collection from the pericardial cavity, directing it into the reservoir. The collected blood was then recirculated, mirroring standard clinical practice. The atrial closure was performed 90 min after CPB initiation. A total of 60 min of complete extracorporeal circulation was performed between the atrial incision and atrial closure.

Blood samples were assayed at 6 timepoints for coagulation factors II, VII and X, haematocrit and platelets. First, baseline samples were collected before the operation. Samples were again collected after full-flow CPB was achieved (0 min timepoint) and at 30, 60 and 120 min after full-flow. Samples were finally collected at 60 min after the monkeys were weaned from CPB. Plasma activities of coagulation factors II, VII and X were measured by kit (coagulation factor activity test factor II [F2], VII [F7], X [F10]; SRL, Tokyo, Japan). We measured platelets and haematocrit using an automated haematology analyser (KX-21; SYSMEX, Kobe, Japan). Plasma activities of coagulation factors are indicated as percentages compared to the control plasma in the kits.

Dilutional effects due to CPB priming solution were also taken into consideration. Coagulation factor activities are thus reported both as absolute values and values adjusted for haemodilution according to the formula [8]: adjusted value = absolute value × (haematocrit level before CPB/haematocrit level at the time of measurement).

### Statistical analysis

All data are presented as mean (standard deviation). Unadjusted and adjusted concentrations of each coagulation factor and platelet counts were analysed with linear mixed-effects model (LMM), with time as a fixed-effects factor and monkeys as a random effect by use of the restricted maximum likelihood method, to assess changes over time. We specified a heterogeneous compound symmetry to variance components through time points in the analysis using LMM. We also assessed the normality of residuals and random effects and did not transform all variables. Kenward and Roger's method was applied for small-sample adjustment [9]. Paired *t*-tests were performed to assess changes in variables compared to the baseline, with the Bonferroni method employed to adjust for multiplicity of testing. The percentage change from baseline was defined as (post value − baseline value)/baseline value × 100 (%). A two-sided test was used and *P *<* *0.05 was considered statistically significant. Data management, statistical analyses and figures were done with the R software package (version 4.2.1 for Windows, R Foundation for Statistical Computing) and SAS (version 9.4; SAS Institute, Cary, NC, USA).

## RESULTS

A total of 7 monkeys (*M. fascicularis*) with a mean weight of 5.06 kg (0.80) underwent procedures. Whole data during the experiments are summarized in Table 1 while changes in factor concentrations from the baseline are shown in Supplementary Material, Table S1. Data of individual monkeys are plotted in Supplementary Material, Figs S1–S3.

**Table 1:** Activities of coagulation factors, platelets and haematocrit during cardiopulmonary bypass (*n* = 7)

Measurement	Time						LMEM results
	Before CPB	0 min CPB	30 min CPB	60 min CPB	120 min CPB	1 h after CPB	
Factor II (%)	115.0. (25.6)	91.6 (19.2)	63.8 (12.8)	63.9 (10.7)	67.4 (9.7)	75.1 (13.4)	<0.001*
Factor VII (%)	134.5 (44.6)	80.4 (38.0)	51.3 (16.0)	54.2 (16.7)	61.5 (18.9)	88.3 (28.9)	<0.001*
Factor X (%)	55.3 (11.8)	44.9 (10.9)	28.6 (5.6)	28.6 (6.6)	30.7 (5.2)	34.9 (6.4)	<0.001*
Platelet count (×10^4^/μl)	34.7 (7.9)	33.8 (6.0)	17.1 (5.2)	15.8 (5.9)	14.9 (5.3)	15.7 (6.4)	<0.001*
Haematocrit (%)	36.2 (3.9)	32.3 (3.9)	21.1 (2.3)	21.2 (3.2)	22.0 (1.9)	25.3 (2.0)	<0.001*
Adjusted factor II (%)	115.0 (25.6)	102.7 (20.7)	109.2 (19.9)	109.8 (18.3)	111.2 (19.2)	107.7 (21.9)	0.118
Adjusted factor VII (%)	134.5 (44.6)	89.8 (41.3)	87.7 (25.0)	92.8 (27.0)	101.7 (33.8)	127.4 (47.3)	0.003*
Adjusted factor X (%)	55.3 (11.8)	50.2 (11.2)	48.0 (7.8)	48.7 (8.8)	50.7 (10.5)	50.3 (12.2)	0.079
Adjusted platelet count (×10^4^/μl)	34.7 (7.9)	37.9 (7.1)	29.7 (9.6)	27.1 (10.3)	24.5 (8.8)	22.3 (8.4)	<0.001*

Data are expressed as percentages in comparison to the reference range in the test kit. Each value represents the mean (standard deviation).

CPB: cardiopulmonary bypass; LMM: linear mixed-effects model.

### Concentrations of coagulation factors during cardiopulmonary bypass

**Figure 1:**
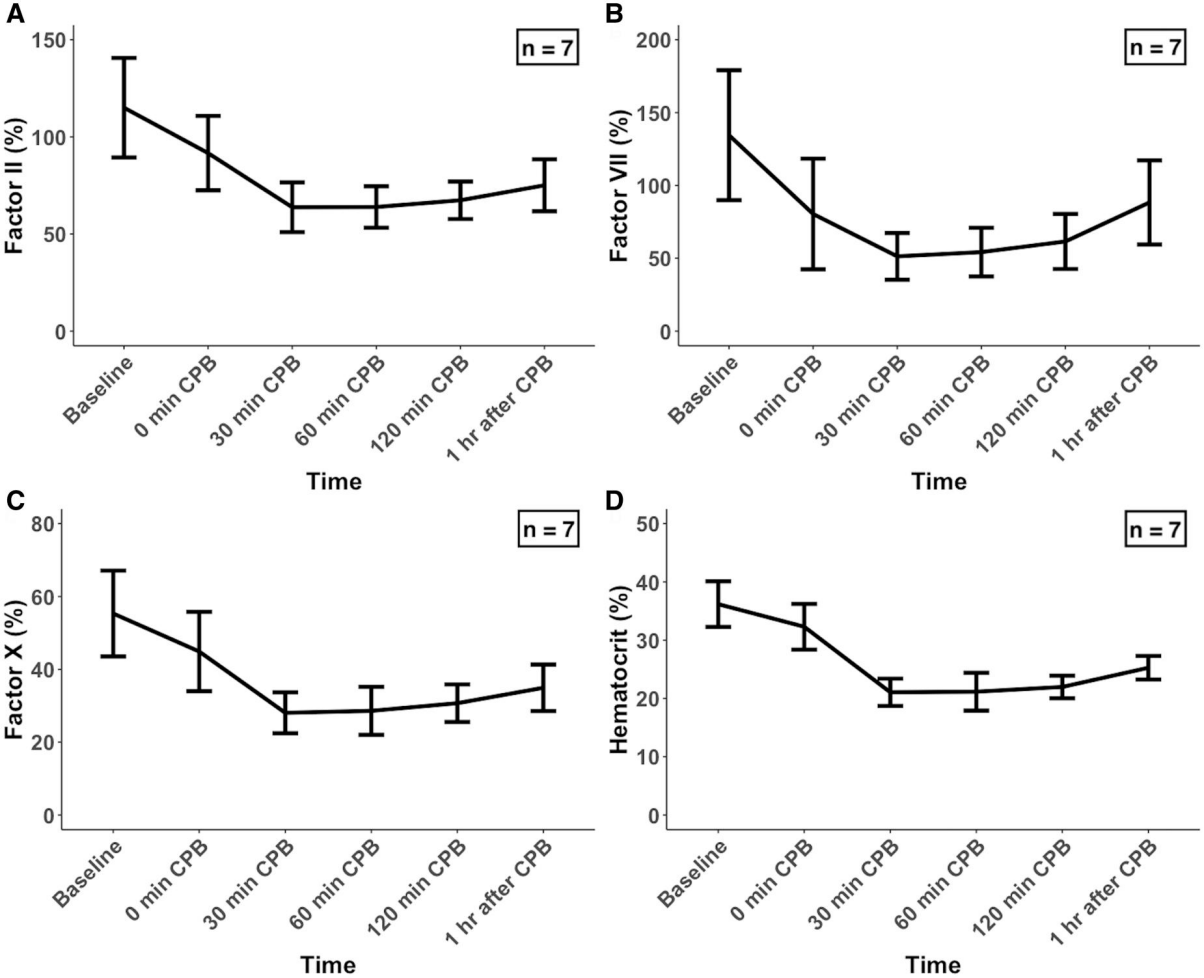
Changes in activities of (**A**) factor II, (**B**) factor VII and (**C**) factor X and (**D**) haematocrit level during CPB. Each value represents the mean (standard deviation). CPB: cardiopulmonary bypass.

Figure 1 and Table 1 show the activities of coagulation factors II, VII and X plus haematocrit concentrations during the experiment. Activities for factors II, VII and X after CPB initiation significantly decreased from the baseline (*P *<* *0.001, *P *<* *0.001 and *P *<* *0.001 by LMM, respectively). In particular, factors II, VII and X had the highest mean decreases from the baseline [44.2% (5.0) (*P *<* *0.001), 61.4% (4.3) (*P *<* *0.001) and 49.0% (3.7) (*P *<* *0.001), respectively] at 30 min following CPB initiation. The activity levels of each factor slightly increased after 30 min. Similar to the profiles for the 3 coagulation factors, the haematocrit concentration decreased after CPB initiation (*P *<* *0.001 by LMM), with the largest mean reduction of 41.7% (4.5) (*P *<* *0.001) from baseline observed at 30 min after CPB initiation.

**Figure 2:**
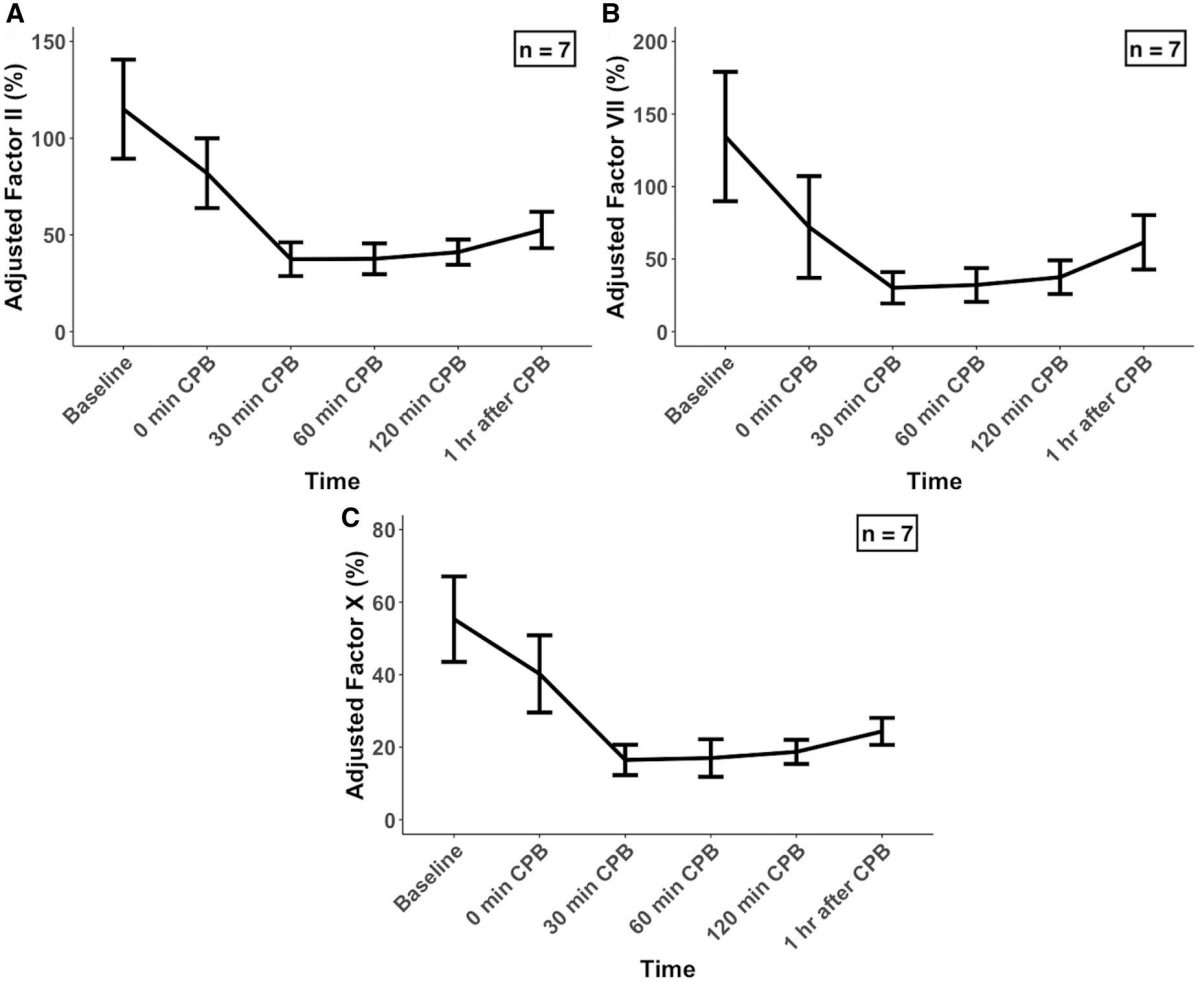
Changes in activities of (**A**) adjusted factor II, (**B**) adjusted factor VII and (**C**) adjusted factor X during CPB. Each value represents the mean (standard deviation). CPB: cardiopulmonary bypass.

To account for haemodilution, the values for coagulation factors were adjusted to the haematocrit levels as previously reported (Table 1) [8]. After normalization for haemodilution, no major changes in factor II and X activities were observed (Fig. 2A and C). Meanwhile, the normalized concentrations for factor VII showed a significant reduction (*P *=* *0.003 by LMM), with a mean 33.5% (9.8) (*P *<* *0.01) drop from baseline at 30 min after CPB initiation (Fig. 2B). Similar to the unadjusted results, normalized concentrations for factor VII increased slightly after the end of CPB.

### Platelet counts during cardiopulmonary bypass

**Figure 3:**
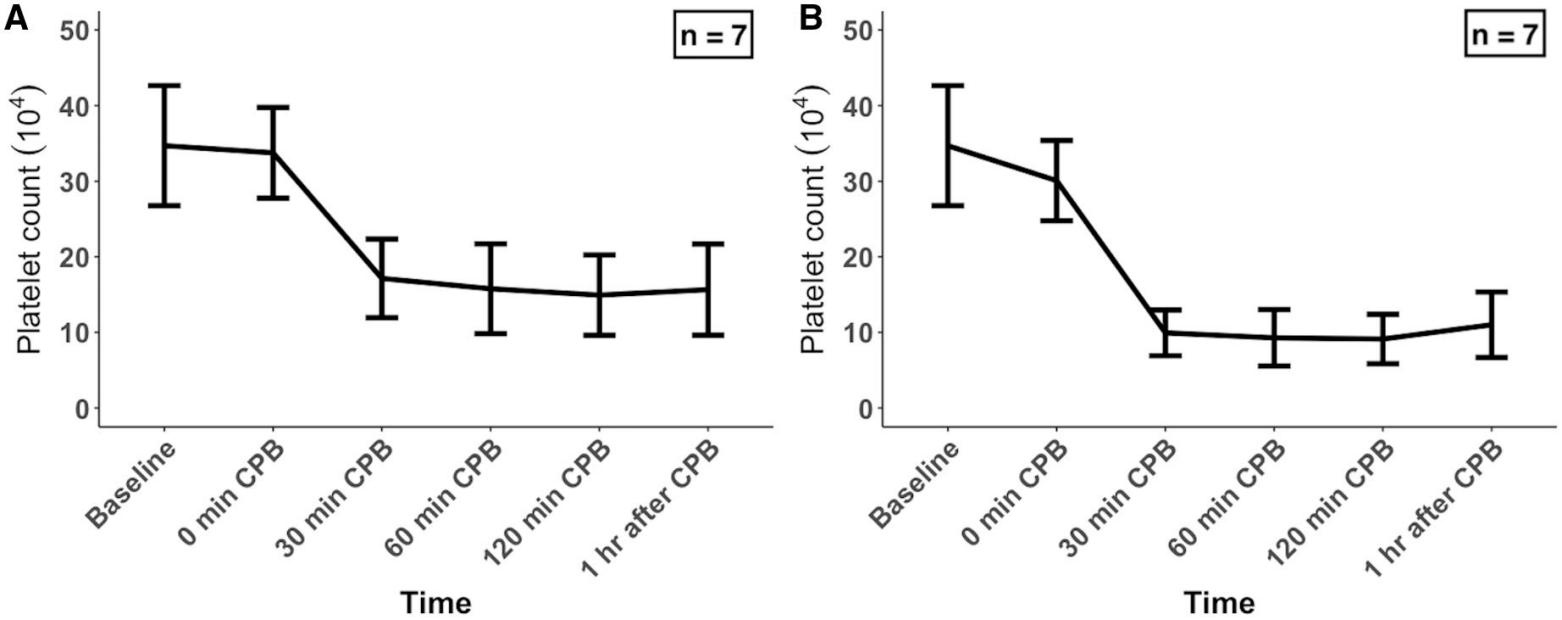
Changes in (**A**) platelet counts and (**B**) adjusted platelet counts during CPB. Each value represents the mean (standard deviation). CPB: cardiopulmonary bypass.

Characterization of platelet counts over time during CPB is shown in Fig. 3. Following the initiation of CPB, the platelet counts significantly decreased during CPB (*P *<* *0.001 by LMM) by a mean of 57.3% (9.5) (*P *<* *0.001) (Fig. 3A). This drop in platelet count was also observed after adjusting for haemodilution (*P *<* *0.001 by LMM), with a mean 37.1% (13.0) reduction (*P *<* *0.001) at the final timepoint compared to the baseline (Fig. 3B).

## DISCUSSION

Our study reproducibly demonstrated decreasing trends in activities of factors II, VII and X following CPB circulation in an established primate model. After normalization of values to account for haemodilution using haematocrit values, such trends became less prominent. However, the findings from this study are useful for characterizing the effects of extended mechanical pumping on haemorrhage risk due to decreases in intrinsic and extrinsic coagulation factors.

Our primate model underwent 120 min of CPB with an additional right atrial incision. Although this model involved far less-invasive procedures compared to actual clinical practice, we observed significant changes indicating coagulation factor consumption and haemodilution. Previous clinical studies that measured the activities of coagulation factors II, VII or X during CPB are summarized in Table 2 [8, 10–18]. Coagulation factor activities before dilution adjustments in our experiment are in line with the literature but, with only 1 other study reporting haemodilution-normalized factor values, more work is needed to fully elucidate the trend with respect to time after CPB initiation [8]. A study by Ternström *et al.* [8] reported adjusted factor VII activity levels to remain unchanged following CPB. However, careful interpretation is necessary as the post-CPB factor values reported in their study were measured 2 h after CPB termination [8]. By then, the regeneration of coagulation factors may have occurred and such a phenomenon could have affected their results [8, 19]. Both unadjusted and adjusted concentrations of factor VII increased 1 h following the termination of CPB and this phenomenon could be attributed to such rapid regeneration by the liver [19]. Additionally, clinical data regarding coagulation factors are prone to variable factors, including intraoperative transfusion and various types of operations. Of 10 previously published studies reporting coagulation factor levels during CPB, only 2 studies reported data from a single type of operation [8, 14]. Therefore, our experimental model provides valuable data in a standardized setting regarding the ‘pure’ influence of CPB on coagulation factors.

**Table 2:** Previous clinical studies reporting concentrations of coagulation factors during cardiopulmonary bypass

Author	Year	HD	*n*	Age (years)	CPB time (min)	Operations	Factor II (%)		Factor VII (%)		Factor X (%)	
							Preop	Postop	Preop	Postop	Preop	Postop
Ternström *et al.*	2010	Yes	57	65 (7)	72 (27)	On-pump CABG	108 (12)	99 (13)	121 (31)	124 (32)	104 (18)	91 (18)
Milam *et al.*	1981	No	75	69 (12)	97	N/A	81 (14.2)	71 (13.3)	94 (31.2)	79 (24.4)	83 (18.7)	71 (15.8)
Harker *et al.*	1980	No	21	55	183	On-pump CABG, ASD, VSD	72 (6.1)	44 (4.3)	109 (11)	58 (4.5)	81 (8.0)	53 (8.3)
Kern *et al.*^a^	1992	No	30	9 days	N/A	TGA, Norwood, TOF, IAA, Ao Atresia, VSD, Truncus	56 (14)	32 (10)	55 (13)	29 (8)	53 (13)	33 (10)
Ootaki *et al.*^a^	2002	No	7	7.12 (2.42)	81.9 (32.9)	ASD, VSD	N/A	N/A	82.9 (17.7)	34.4 (7.0)	63.7 (23.8)	31.3 (5.5)
Mittermayr *et al.*	2009	No	16	65 (10)	118 (42)	On-pump CABG	92 ± 12	51 ± 1	82 ± 16	59 ± 15	81 ± 13	45 ± 9
Karkouti *et al.*	2010	No	101	69 (12)	138 (41)	On-pump CABG, valve replacement	90 (20)	50 (10)	N/A	N/A	90 (20)	50 (20)
Schols *et al.*	2010	No	30	N/A	N/A	N/A	N/A	N/A	N/A	N/A	83 (13)	53 (13)
Coakley *et al.*	2011	No	77	69 (29–89)	132 (99–185)	On-pump CABG, valve replacement, ascending aortic aneurysm repair, redo	88 (78–98)	43 (35–53)	94 (79–105)	55 (47–69)	88 (77–97)	41 (30–52)
Solomon *et al.*	2013	No	32	61.1 (32–88)	N/A	Thoracoabdominal aortic aneurysm surgery, aortic valve and ascending aorta replacement	96 (81–105)	48 (42–57)	N/A	N/A	N/A	N/A

Data are expressed as mean (standard deviation) or median (interquartile range).

a Studies targeting paediatric populations.

ASD: atrial septal defect repair; TGA: transposition of the great arteries; TOF: tetralogy of Fallot; IAA: interrupted aortic arch;  CABG: coronary artery bypass graft; CPB: cardiopulmonary bypass; HD: adjustment for haemodilution; N/A: not available; VSD: ventricular septal defect repair.

We believe that the observed decreases in adjusted factor VII activity demonstrate the activation of the extrinsic coagulation cascade. Interestingly, adjusted factor VII decreased mainly between the baseline and the initiation of full-flow CPB, likely due to massive thrombotic stimuli from tissue factors aspirated from the surgical field, mediated by the extrinsic cascade [3]. Tissue factors may activate as soon as 15 min after the induction of CPB [20], coupled with continuous activation and consumption that could result in such a trend during CPB flow. The sustained concentrations of factor VII during CPB flow could be attributed to rapid regeneration to maintain homeostasis with the equally rapid consumption. The activation of the intrinsic cascade from the blood–machine interface may have mediated the changes observed in factors II and X [1]. However, the temporal changes in adjusted factor X during CPB, as well as factor II, were not as evident as factor VII. The administration of heparin prior to the establishment of the CPB circuit may be responsible for the difference observed among different coagulation factors.

Heparin, which binds to antithrombin III to inhibit factor II formation, is administered to prevent clotting during cardiac operations using CPB [21]. Heparin inhibits factors II and X to produce anticoagulant effects, which may have attenuated activation and consumption [21], possibly leading to observed changes in adjusted factors II and X in our experiment. The role of heparin in the extrinsic pathway has received recent attention and our group previously reported that heparin decreases factor II generation via the release of tissue factor pathway inhibitor which, in turn, directly inhibits the activities of both tissue factor and factor VII [22]. Such a mechanism may have reduced the consumptive coagulopathy potential of factor VII, blunting our results. In addition to heparin modulation of factor VII effects, the observed decrease in factor VII could have been influenced by other mechanisms. Heparin was found to lower the concentration of activated factor VII in a prior study in healthy subjects [23] without CPB circulation. Further clinical studies exploring the association between heparin and coagulation factors during extended CPB are warranted.

In this study, we demonstrated that CPB, even with less-invasive cardiac procedures, could lead to coagulopathy. We thus postulate that invasive cardiac procedures would further exacerbate consumptive coagulopathy from the extrinsic coagulation cascade due to enhanced tissue factor release. This hypothesis is supported by a previous report that linked aortic replacement surgeries with increased activation of coagulation cascades in comparison to aortic valve replacement surgery [24]. Furthermore, it has also been demonstrated that off-pump coronary artery bypass graft operations result in less consumptive coagulopathy in comparison to on-pump coronary artery bypass graft operations [25].

Platelets are a critical factor in bleeding management as decreased platelet counts largely contribute to post-CPB coagulopathies in addition to platelet dysfunction [11]. With regard to platelet counts, our findings were in line with the current literature since we observed decreases of up to 50% during CPB [2]. A previous study reported less platelet activation and consumption with off-pump versus on-pump coronary artery bypass grafts, demonstrating the role of CPB in platelet dysfunction during cardiac operations [26]. In contrast to coagulation factors, no increase following CPB was observed in platelet counts. Since it takes ∼5 days for platelet counts to return to preoperative baseline levels [27], our observed lag in replenishment was clinically relevant.

### Limitations

This study has several limitations. First, our experimental primate model may not directly translate to humans. However, compared to other animals (e.g. pigs), blood components of *M. fascicularis* are similar to humans, including coagulation factors and other biomarkers related to clotting time [28]. Although there has been a study reporting the concentration profile of coagulation factor VII during CPB in pigs [29], the coagulative properties of blood are considerably different between humans and pigs, rendering those results impractical for clinical settings [30]. Another limitation of this study is the small sample size, necessitated by animal availability and ethics (*n* = 7). Third, our experimental model only involved an atrial incision in addition to CPB, which may not truly be reflective of clinical settings. However, further invasive procedures were deemed a high risk to the animals and proscribed by ethical guidelines. In addition, as our experiment measured coagulation factors only in the common and the extrinsic pathways, data regarding the intrinsic pathway (coagulation factors VII, IX, XI and XII) could have provided additional clarity. Finally, our primate model did not include cardiac arrest during the experiment. Although we attempted to establish such a primate model to better represent clinical situations, the mortality rate following cardiac arrest was high, especially due to lack of blood transfusion. Thus, our primate model excluded cardiac arrest through cardioplegia.

## CONCLUSION

We investigated the effect of CPB on coagulation factor activities in an *in vivo* primate model and observed a decreasing trend of coagulation factor activities during CPB. Following normalization with haematocrit values to account for haemodilution, such differences became less prominent although still statistically significant for coagulation factor VII. We postulate that consumptive coagulopathy and haemodilution play a major role in postoperative coagulopathies during extended CPB times, requiring vigilant countermeasures.

## Supplementary Material

ivad194_Supplementary_Data

## Data Availability

The data underlying this article are available in the article and in its online supplementary material.

## ABBREVIATIONS

CPB: Cardiopulmonary bypass
LMM: Linear mixed-effects model
