# Supplementary material for: Effect of cardiopulmonary bypass on coagulation factors II, VII and X in a primate model: an exploratory pilot study
Source: Interdiscip Cardiovasc Thorac Surg. 2023 Nov 28;37(6):ivad194. doi: 10.1093/icvts/ivad194 (PMC10701202; doi:10.1093/icvts/ivad194)
Supplement: ivad194_Supplementary_Data [file ivad194_supplementary_data.docx]

**Supplementary Materials**

Shimoda T, Liu C, et al. “Effect of cardiopulmonary bypass on coagulation factors II, VII, and X in a primate model”

**Supplementary Table 1:** (Page 2)

Results of paired t-test for each variable compared to the baseline (*P* values).

**Supplementary Figure 1:** (Page 3)

Concentration-time profiles for (A) factor II, (B) factor VII, (C) factor X, and (D) hematocrit during CPB (Lines with color represent data from individual monkeys). CPB=cardiopulmonary bypass

**Supplemental Figure 2:** (Page 4)

Concentration-time profiles for adjusted (A) factor II, (B) factor VII, and (C) factor X during CPB (Lines with color represent data from individual monkeys). CPB=cardiopulmonary bypass

**Supplemental Figure 3:** (Page 5)

Concentration-time profiles for (A) platelets and (B) adjusted platelets during CPB (Lines with color represent data from individual monkeys). CPB=cardiopulmonary bypass

**Supplemental Table 1**

Results of paired t-test for each variable compared to the baseline (*P* values).

| **Time vs Baseline** | **Factor II** | **Factor VII** | **Factor X** | **Platelets** | **Hematocrit** | **Adjusted Factor II** | **Adjusted Factor VII** | **Adjusted Factor X** | **Adjusted Platelets** |
| --- | --- | --- | --- | --- | --- | --- | --- | --- | --- |
| 2 | <0.05 | <0.05 | <0.05 | 1 | <0.05 | 0.190 | <0.05 | 0.144 | <0.05 |
| 3 | <0.05 | <0.05 | <0.05 | <0.05 | <0.05 | 1 | <0.05 | <0.05 | 0.066 |
| 4 | <0.05 | <0.05 | <0.05 | <0.05 | <0.05 | 1 | <0.05 | 0.077 | 0.065 |
| 5 | <0.05 | <0.05 | <0.05 | <0.05 | <0.05 | 1 | <0.05 | 0.137 | <0.05 |
| 6 | <0.05 | <0.05 | <0.05 | <0.05 | <0.05 | 0.504 | 1 | 0.289 | <0.05 |

**Supplemental Figure 1:**

Concentration-time profiles (%) for (A) factor II, (B) factor VII, (C) factor X, and (D) hematocrit during CPB (Lines with color represent data from individual monkeys). CPB=cardiopulmonary bypass


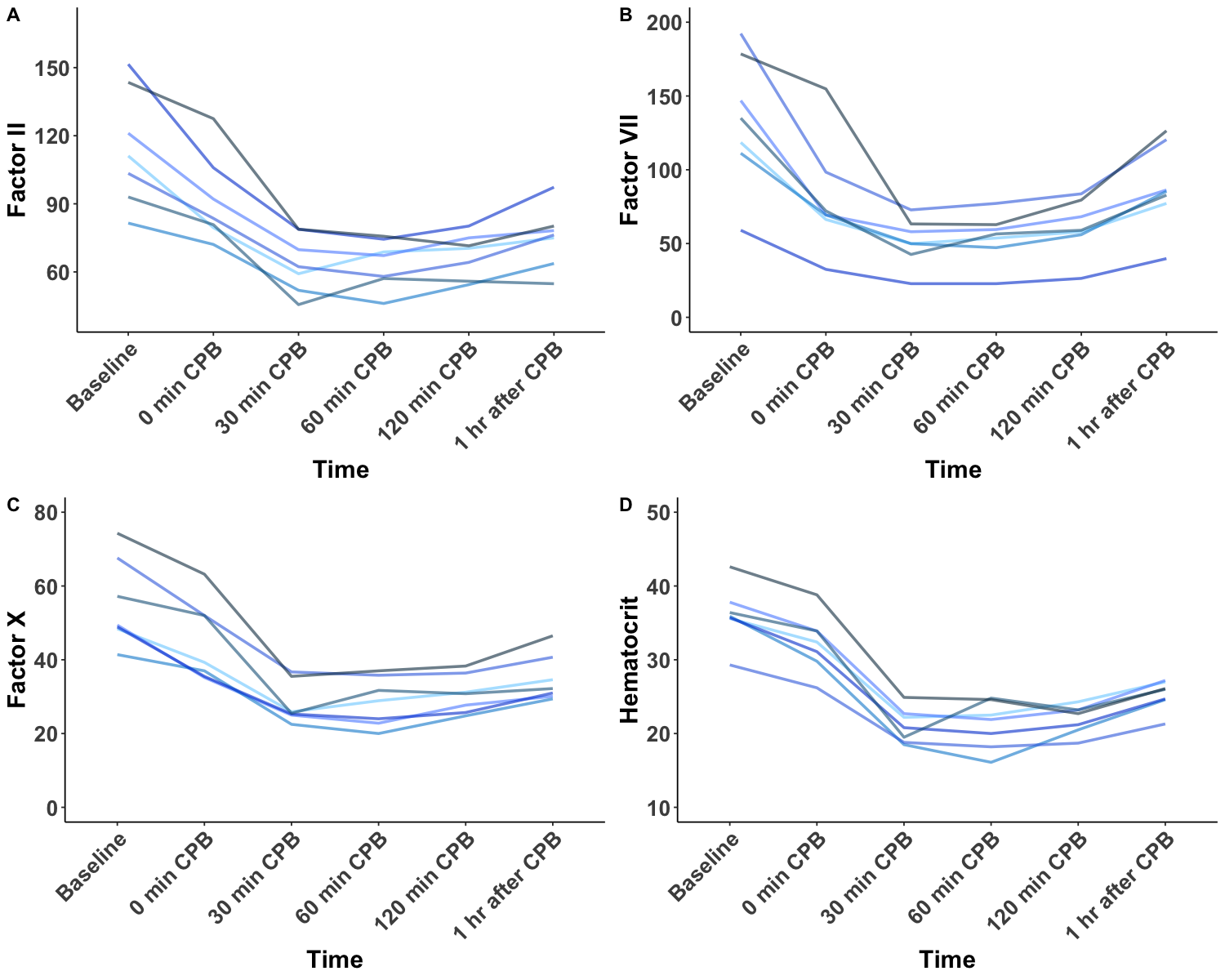


**Supplemental Figure 2:**

Concentration-time profiles (%) for adjusted (A) factor II, (B) factor VII, and (C) factor X during CPB (Lines with color represent data from individual monkeys). CPB=cardiopulmonary bypass

**
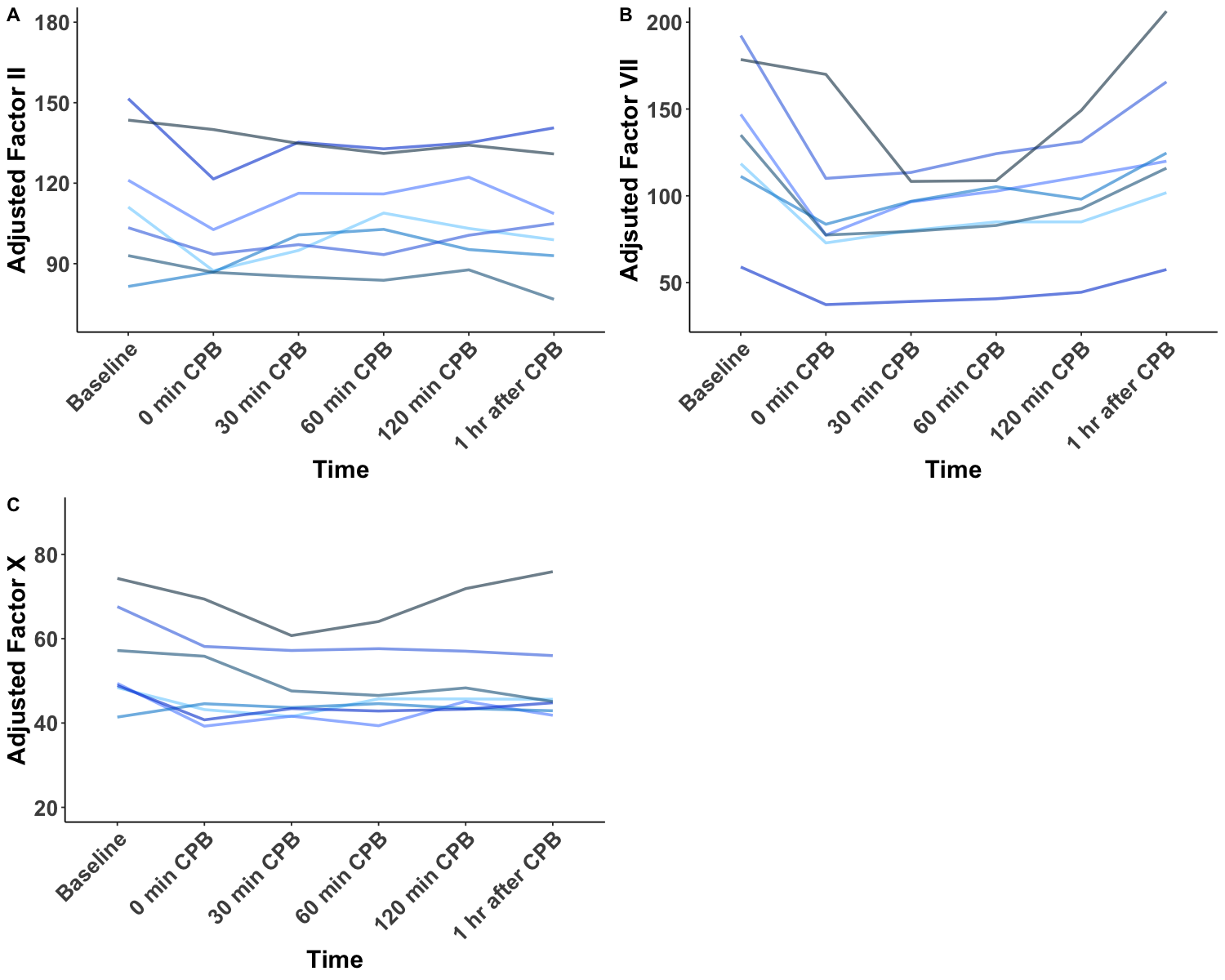
**

**Supplemental Figure 3:**

Concentration-time profiles for (A) platelet counts and (B) adjusted platelet counts during CPB (Lines with color represent data from individual monkeys). CPB=cardiopulmonary bypass

**
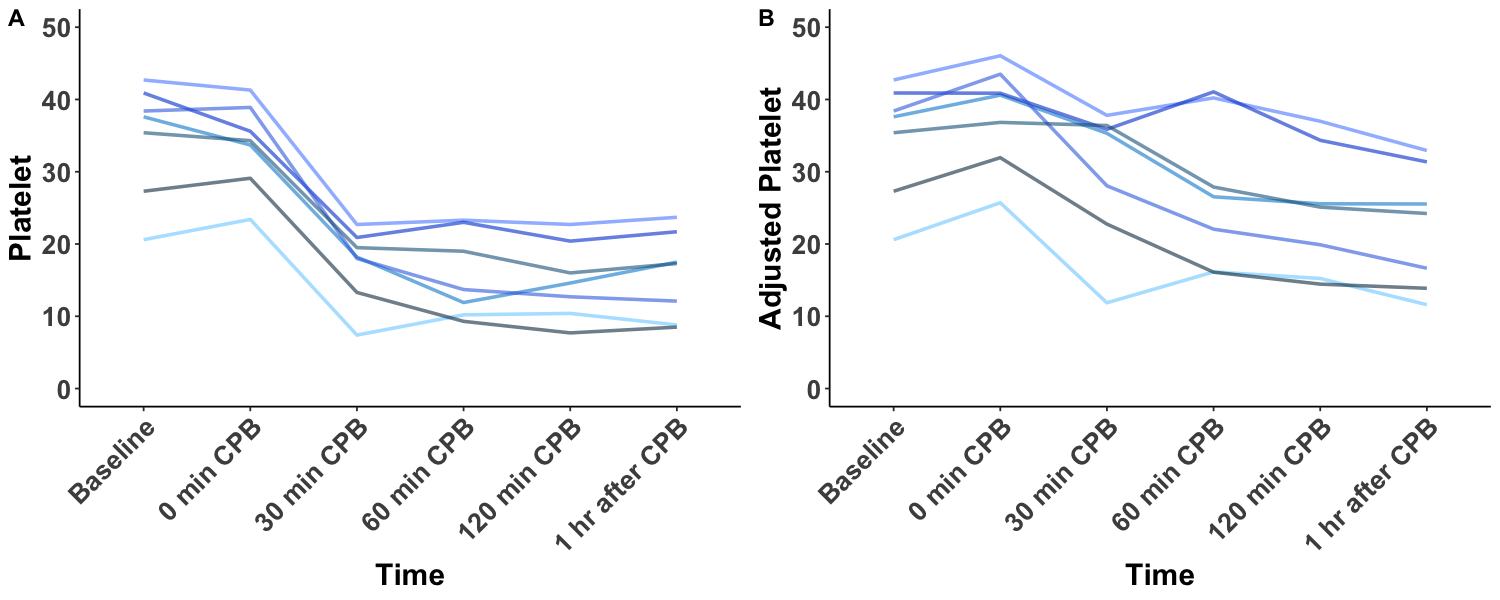
**
